# Complexity of human death: its physiological, transcriptomic, and microbiological implications

**DOI:** 10.3389/fmicb.2023.1345633

**Published:** 2024-01-12

**Authors:** Gulnaz T. Javan, Kanhaiya Singh, Sheree J. Finley, Robert L. Green, Chandan K. Sen

**Affiliations:** ^1^Department of Physical and Forensic Sciences, Alabama State University, Montgomery, AL, United States; ^2^Department of Surgery, School of Medicine, McGowan Institute for Regenerative Medicine, University of Pittsburgh, Pittsburgh, PA, United States

**Keywords:** physiology, gene expression, human postmortem microbiome, human decomposition, artificial intelligence

## Abstract

Human death is a complex, time-governed phenomenon that leads to the irreversible cessation of all bodily functions. Recent molecular and genetic studies have revealed remarkable experimental evidence of genetically programmed cellular death characterized by several physiological processes; however, the basic physiological function that occurs during the immediate postmortem period remains inadequately described. There is a paucity of knowledge connecting necrotic pathologies occurring in human organ tissues to complete functional loss of the human organism. Cells, tissues, organs, and organ systems show a range of differential resilience and endurance responses that occur during organismal death. Intriguingly, a persistent ambiguity in the study of postmortem physiological systems is the determination of the trajectory of a complex multicellular human body, far from life-sustaining homeostasis, following the gradual or sudden expiry of its regulatory systems. Recent groundbreaking investigations have resulted in a paradigm shift in understanding the cell biology and physiology of death. Two significant findings are that (i) most cells in the human body are microbial, and (ii) microbial cell abundance significantly increases after death. By addressing the physiological as well as the microbiological aspects of death, future investigations are poised to reveal innovative insights into the enigmatic biological activities associated with death and human decomposition. Understanding the elaborate crosstalk of abiotic and biotic factors in the context of death has implications for scientific discoveries important to informing translational knowledge regarding the transition from living to the non-living. There are important and practical needs for a transformative reestablishment of accepted models of biological death (i.e., artificial intelligence, AI) for more precise determinations of when the regulatory mechanisms for homeostasis of a living individual have ceased. In this review, we summarize mechanisms of physiological, genetic, and microbiological processes that define the biological changes and pathways associated with human organismal death and decomposition.

## 1 Introduction

The study of death is unmatched in the span of scientific disciplines it incorporates, including antemortem studies of evolutionary, genetic, and molecular forces that shape the progression toward death to postmortem studies that elucidate human decomposition. Death is the irremediable state of cellular damage that ensues in response to the cessation of cardiac, brain, and respiration functions. It is the completion of a biological trajectory that commences at conception and culminates with termination of all bodily functions with no possibility of recovery or resuscitation. The processes of death occur in coordinated stages that are differentiated by agonal suppression and subsequent stopping of all essential functions. Death can result from pathological causes such as disease, genetically programmed aging, or unnatural causes such as accidental, suicidal, and homicidal injuries.

Death is characterized by declines in biological resilience, which is an incapability of a body to recover, and is a key indicator of the susceptibility to limited longevity that ultimately leads to death (Liu et al., 2018). As a person ages, the ability a body to fully return to the coordinated physiological processes of homeostasis after deviation from normal steady state or after damage due stressors or adverse health events slows compared to its younger state (Ukraintseva et al., 2021). In this review, we recapitulate the coordinated mechanisms of the physiological and pathological processes that engage in crosstalk of coordinated biological changes and pathways that culminate to human organismal death and decomposition.

## 2 Physiological definition of death

Human death is the irreversible and permanent cessation of all vital biological functions and processes that sustain life in an individual. Physiological death of the human occurs when the vital organs of the body lose their functions in a way that results in irreversible loss of (i) circulatory and respiratory functions, or (ii) all functions of the entire brain, including the brain stem. In 2020, the World Brain Death Project provided comprehensive clinical guidance on methods to diagnose brain death or death by neurologic criteria (BD/DNC) typified by different clinical conditions and settings (Greer et al., 2020). The guidance was composed based on an assessment of literature along with the expert opinions of a large multidisciplinary panel of international professionals from various fields. The recommendations necessitate the completion of a prior neurologic evaluation for BD/DNC that establishes the complete and irreversible loss of all brain function. Further, the recommendations require an exclusion of conditions that may mimic the clinical examination of BD/DNC as well diseases that lead to the misperception of BD/DNC. Lastly, the Project listed eight clinical tests for physical manifestations of BD/DNC that conclude BD/DNC (Greer et al., 2020).

During the natural process of dying, vital organ functions enter a phase of progressive decline (Adeyinka and Bailey, 2023). For example, the process of digestion slows down and the digestive track loses moisture. Processes such as chewing, swallowing, and elimination become painful. Slowing of the circulatory system manifests as mottling or pooling of blood at the underside of the body. Breathing becomes more sporadic and shallower often associated with rattling sound as air travels through mucus filled respiratory passage. Such labored breaths are caused by an abnormal pattern of brainstem reflex caused by inadequate oxygenation. Agonal breathing or agonal respirations describe complicated breathing that often sounds like snoring, snorting, gasping, or labored breathing. The person appears to be choking or having an involuntary gasp reflex. The dying person often sleeps more and may talk less although hearing senses remain functional. The Uniform Determination of Death Act (UDDA) provides a legal framework for determining death, specific protocols and guidelines for diagnosing death, especially brain death, may vary by jurisdiction and medical institution (Lewis, 2022). This act primarily serves as a model law for states to adopt, and the exact wording and application of the law can differ from one state to another. There are five manners of death: natural, accident, suicide, homicide, and undetermined (and pending). Recently, death is also being classified based on the terminal state of the patient just before the cessation of the life based on Hardy Scale (Hardy et al., 1985; Dai et al., 2021).

### 2.1 Death classification based on the four-point hardy scale

1. Violent fast death. Cases belonging to this type of death range from accidental, homicidal, suicidal, or blunt trauma. The terminal phase of life is estimated at less than 10 min.

2. Fast death of natural causes. These are reasonably healthy people who suffered sudden, unexpected death, after a terminal phase estimated at less than 1 h. Most of these cases die at home. Death is attributed to the progression of a disease or pre-existing condition, and which is not attributable to any outside action or force. Sudden death from a myocardial infarction remains the most frequent cause of fast death type.

3. Intermediate death. This group consists of patients who were ill, generally admitted to the hospital, but whose deaths are unexpected. The terminal phase of this type of death ranges from 1 to 24 h. They could neither be classified as fast deaths (above two categories) nor as slow deaths (below mention category).

4. Slow death. These are deaths after a long illness with a prolonged terminal phase (greater than 1 day). Death is not unexpected, and patients die typically from cancers, cerebrovascular disease, or chronic pulmonary disease. Most of these deaths happen in the hospital.

0. Ventilator Case: These are subjects who are on a ventilation machine immediately before death.

### 2.2 Biochemical changes associated with death

Death results in massive biochemical changes in the body acutely caused by inadequacies in circulating oxygen. Such limitation results in a switch of metabolism from aerobic citric acid cycle to anaerobic (Sawyer et al., 1988). These biochemical changes lead to chemical indicators useful in accurately determining postmortem interval. For example, postmortem fall in blood pH is a result of accumulation of metabolites such as lactate (C_3_H_5_O_3_^–^) and formate (HCO_2_^–^) (Donaldson and Lamont, 2013). Furthermore, anaerobic glycolysis in postmortem tissue results in an increase in the concentration of NADH. Sawyer et al. (1988) demonstrated that cardiac blood taken from human remains after death becomes acidic (pH 7.0 to 5.5) by 20 h postmortem. In rat corpses cardiac blood pH dropped from 7.35 (antemortem) to 5.5 after 96 hrs postmortem (Sawyer et al., 1988). These metabolic changes postmortem are results of: (i) the agonal period of severe hypoxia; and (ii) the distribution of easily diffusible biochemicals between cellular and extracellular compartments (Donaldson and Lamont, 2013). Hence the analysis of biochemical markers postmortem could be useful to estimate the duration of agonal period and to define the cause of death (Rosato et al., 2021).

Some of the biochemical markers currently being used to evaluate agonal period are: (i) C-reactive protein and acute phase markers to diagnose the presence of infection or inflammation to differentiate between acute and non-acute death (Astrup and Thomsen, 2007); (ii) blood ferritin serves as a postmortem biomarker used in estimating the agonal period after trauma (Ondruschka et al., 2018); and (iii) Liver-Type Fatty Acid-Binding Protein (L-FABP) and 8-Hydroxy-2-Deoxyguanosine (8-OHdG): urinary detection of L-FABP and 8-OHdG are used as potential biomarkers for the estimation of agonal duration (Kashiwagi et al., 2013). These biomarkers are not influenced by postmortem time interval and are comparatively more stable in the urine than in the blood (Kashiwagi et al., 2013). As caution, it is important to note that pre-existing disorders during life such as diabetic ischemic diseases, environmental factors during death, postmortem tissue degeneration, and the analytical approach for the estimation of biochemicals may serve as confounding factors in biomarker detection (Maeda et al., 2010; Rosato et al., 2021).

Recent advances in molecular biology including the emergence of single cell genomics enable the investigation of the genetic basis of diseases associated with the death type of the individual. This has led to the emergent notion of “molecular autopsy” (Rosato et al., 2021). Systematic postmortem quantitative analysis of mRNA transcripts has the potential to be established as a core part of the molecular pathology for death investigations in forensic pathology, to support and reinforce morphological evidence.

## 3 Gene expression

### 3.1 Gene expression before death

Life originates and concludes in hypoxic conditions in that vertebrates are contained in low oxygen environs prior to the development of the systems of the body. In this low level of blood oxygen, developmental genes are activated and begin to transcribe DNA in the genetic network controlling the early stages of embryogenesis and stem cells differentiation (Assou et al., 2011; Podkalicka et al., 2020). During life, homoeostasis is maintained in healthy human adults by epigenetic networks that coordinate through multiple feedback mechanisms of genetic loci to precisely synchronize gene expression at the precise time and in the appropriate quantities for the sustenance of human life (Emilsson et al., 2008).

Transcriptomic activity is a link between the genome, the proteome, and the cellular phenotype, and drives a cell’s structure and function at any given time. Gene expression is primarily regulated at the level of transcription by varying the production of messenger RNA (mRNA) from genes. Generally, the human transcriptome is the complete expression of mRNA transcripts produced in all tissues. Prior to death, terminally differentiated cells have distinctive patterns of gene expression. Research has established that up to 10% of mRNA transcripts are programmed by tissue-enriched genes, with some genes being enhanced to such an extent that the mRNA levels in one tissue type are almost five times the maximum concentrations of all other tissues (Uhlén et al., 2015). mRNA transcriptome analysis from a tissue fragment allows the designation of its origin to a specific organ, since each will demonstrate a distinctive pattern of gene expression (Sauer et al., 2017).

### 3.2 Gene expression after death

Tissue specific transcriptome analysis offers insight into the underlying diseases and molecular mechanisms associated with human death. Molecular transcriptomic analyses may help develop important tools that would help determine the cause of death (Bauer, 2007). It should be recognized, however, that unlike DNA, which remains relatively stable over long postmortem periods, RNA is fragile in nature, and sensitive to degradation in a tissue-specific manner (Bauer, 2007). However, recent studies in different mammals have shown that when samples remain properly collected and stored, RNA can remain largely intact even for considerable time periods (Ferreira et al., 2018). As a result, degeneration of RNA and/or the loss of certain RNA transcripts, in terms of rapidity and temporal correlation, is now being utilized as a possible indicator of postmortem ischemia (Poór et al., 2016; Scrivano et al., 2019). This correlation is possible because after human death, RNA degradation by cellular ribonucleases (RNases), bacteria, or other environmental contamination depends not only on time but also on other extrinsic factors such as cause of death and environmental conditions (Sampaio-Silva et al., 2013). Traditionally, real-time quantitative polymerase chain reaction (qRT-PCR) has been a universally accepted method for the detection of mRNA, ribosomal RNA (rRNA), and microRNA (miRNA), for the estimation of postmortem interval (PMI). However, appropriate data normalization is considered crucial to nullify the variations in RNA levels due to sample processing. In addition, following practical considerations as advocated by Vennemann and Koppelkamm (2010a,b) should be adopted: (i) careful selection of sample set; (ii) high sample size per group; (iii) accuracy in handling and processing; (iv) careful interpretation of quantitative gene-expression data; and (v) adequate normalization strategy.

Over the past few years, RNA-Sequencing (RNA-seq) has gained increasing popularity and supplanted RT-PCR as the primary high-throughput choice for quantifying the whole transcriptome in postmortem samples (Hanson and Ballantyne, 2017; Xu et al., 2017; Zhu et al., 2017; Javan et al., 2020; Lindholm Carlström et al., 2021). RNA-Seq technology is based on next-generation sequencing that directly determines the complementary DNA (cDNA) sequence in postmortem samples. Advances in large-scale RNA-Seq data analyses have already led to a detailed atlas of the human postmortem tissue transcriptome (Melé et al., 2015). The Genotype-Tissue Expression (GTEx) project provides an opportunity to systemically investigate the genome-wide patterns of change in gene expression levels associated with PMI in various human postmortem tissues (Melé et al., 2015). This project was initiated by the National Institutes of Health (NIH) Common Fund to determine how genetic variation affects normal gene expression in human tissues, and thus ultimately informs the study of human disease. Samples were collected from 54 non-diseased tissue sites across nearly 1000 human donors. High-quality postmortem tissues were used to perform RNA-seq analyses. Accurate postmortem intervals were recorded for most samples, ranging from one to 27 h (Zhu et al., 2017). The GTEx database is therefore well suited for transcriptome research into how time of death affects the gene expression across diverse postmortem human tissues.

**FIGURE 1:**
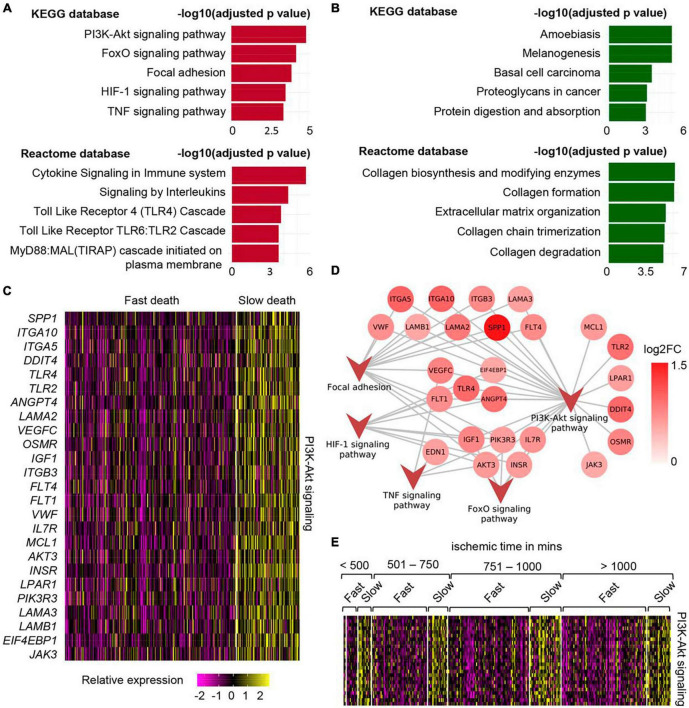
Upregulation of PI3K-Akt mTOR signaling in human post-mortem skin samples from subjects with longer terminal phase (slow death). **(A)** Top 5 upregulated pathways in skin cells of slow death-type cases compared to fast death cases using KEGG (upper) and Reactome (lower) databases. **(B)** Top 5 downregulated pathways in skin cells of slow death-type cases compared to fast death-type cases using KEGG (upper) and Reactome (lower) databases. **(C)** Heatmap showing upregulated genes involved in PI3K-Akt signaling pathway in slow death-type cases. **(D)** Network representing the identified genes in PI3K-Akt signaling pathways and their involvement (edges) in other upregulated pathways, i.e., FoxO signaling pathway, focal adhesion, HIF-1 signaling pathway and TNF signaling pathway. **(E)** Heatmap representing PI3K-Akt signaling pathway genes [panel **(C)**] split by post-mortem ischemic time bins. Figure adapted from Abouhashem et al. (2023), bioRxiv.

In a work recently reported by us, the effect of different durations of terminal phase of human life on postmortem changes in cellular gene expression was investigated using RNA-seq data from 701 human skin samples from the GTEx database (Abouhashem et al., 2023). In depth mining of bulk RNA sequencing data followed by validation using single nuclei sequencing data demonstrated that in human post-mortem skin, slow-death (longer terminal phase of dying) was associated with a more robust induction of survival pathways (e.g., PI3K-Akt, mTOR, and FoxO signaling) (Figure 1 and Table 1). Interestingly, this upregulation of survival pathways was independent of sex of the individual or the duration of death-related post-mortem tissue ischemia. However, in depth investigation is warranted to explain such gene expression alteration and its downstream functional significance associated with the nature of the terminal phase of human life. Detailed characterization of distinct transcriptional programs thus identified might reveal novel directions in the domain of regenerative medicine (Abouhashem et al., 2023).

**TABLE 1:** Table listing the genes that were used to calculate PI3K-Akt signaling pathway score in slow death-type cases.

Gene	Adipose	Endothelial	Epithelial	Fibroblast	Immune	Other
*SPP1*	0.00	0.00	0.00	0.00	0.18	0.03
*ITGA10*	0.20	0.63	0.08	0.36	0.09	0.13
*ITGA5*	1.03	1.53	0.09	1.47	0.17	0.79
*DDIT4*	2.46	1.50	0.71	1.41	0.66	1.53
*TLR4*	0.00	0.17	0.00	0.16	0.10	0.00
*TLR2*	0.06	0.02	0.04	0.00	1.07	0.06
*ANGPT4*	0.00	0.02	0.01	0.03	0.00	0.50
*LAMA2*	1.07	0.95	0.27	4.67	1.38	1.25
*VEGFC*	0.00	0.65	0.03	0.77	0.05	0.01
*OSMR*	0.79	0.97	0.52	0.80	0.13	0.86
*IGF1*	0.27	0.20	0.01	0.28	0.03	0.00
*FLT4*	0.00	0.85	0.00	0.02	0.07	0.04
*FLT1*	0.27	2.36	0.05	0.02	0.62	0.71
*VWF*	0.15	2.54	0.06	0.10	0.23	0.31
*IL7R*	0.00	0.02	0.01	0.17	1.61	0.05
*MCL1*	0.50	0.26	0.85	0.73	0.83	1.29
*AKT3*	0.95	3.06	0.69	3.13	2.28	1.69
*INSR*	2.18	1.40	1.91	2.38	1.68	1.73
*LPAR1*	0.00	0.16	0.10	2.87	0.50	0.39
*PIK3R3*	0.00	0.48	0.03	0.02	0.11	0.13
*LAMA3*	0.62	1.19	2.59	0.69	1.07	2.14
*LAMB1*	1.53	0.15	1.14	2.28	0.54	1.37
*EIF4EBP1*	0.45	0.08	0.15	0.01	0.25	0.34
*JAK3*	0.00	0.28	0.05	0.05	0.30	0.05

Each gene expression average of genes used to calculate PI3K scores within different skin compartments (adipose, endothelial, epithelial, fibroblast, immune and rest other) in the single nucleus RNA-seq data (values expressed in log2 fold change). Table adapted from Abouhashem et al. (2023), bioRxiv.

During life, genetic associations and epigenetic modifications to the genome are precisely orchestrate the vast network of genes—but in death, clarity is required to determine if gene expression diminishes gradually or abruptly, and which identifiable genes and pathways are involved (Scott et al., 2020). Most gene activity is extinguished after organismal death. However, several studies have highlighted the efficacy of using RNA-seq to permit the assessment of changes in global gene expression profiles in dying vertebrates.

Postmortem human specimens are valuable resources for gene expression research after death specifically in the understanding the genetic associations into decomposition mechanisms (Highet et al., 2021; Collado-Torres et al., 2023; Thakral et al., 2023). However, there is a long-standing barrier regarding using human tissues in gene expression research due to fidelity of postmortem tissue and postmortem mRNA degradation patterns. Postmortem gene expression is described by the detectable mRNA transcript abundance and includes is all mRNA that are transcribed from the portion of the genome that remains active or is awakened after organismal death (Javan et al., 2015). The rates of decay of mRNA transcripts fluctuate depending on the types of postmortem tissues and their combinatorial gene expression signatures. mRNA transcript abundances are degraded by cellular machinery which involves complex and highly regulated pathways.

The term relative abundances of mRNA transcripts also refer to the array of mRNA quantities from either dying cells or dead cells that exhibit differential mRNA degradation patterns. The absolute mRNA abundance predictably declines with accumulating PMI (Collado-Torres et al., 2023). Additionally, postmortem studies have shown that the rate of decline and the nature of the mRNA transcripts demonstrating degradation reduce in a predictable manner (Bauer, 2007). For example, there is considerable reduction in RNase activity at extended times since death as demonstrated in dehydrated mummified organs and dried blood (Botling et al., 2009; Fordyce et al., 2013). Furthermore, Bauer et al. (2003) reported that mRNA from stored dried blood samples can be used for as long as 15 years. A smaller fraction of mRNA transcripts persists and increases after death, also in a predicable manner. Specific tissue types have demonstrated detectable degrees of proclivity and sensitivity to PMI-related mRNA degradation and have differential PMI-associated genes when compared to other tissues (Zhu et al., 2017).

When studying deceased humans, the change of expression levels of genes, even a minor-fold increase or decrease, is substantial; specific changes occur in postmortem mRNA transcript abundances in a time- and manner of death-dependent method depending on the type of tissue specimens (Hanson and Ballantyne, 2017; Ballard et al., 2020; Javan et al., 2020; Haas et al., 2021). Thus, human sample mRNA degradation patterns have the propensity to be novel molecular biomarkers. Hanson and Ballantyne (2017) developed and investigated a targeted, massively parallel sequencing (MPS) 46-mRNA biomarker panel that included numerous gene targets for the identification of internal organs (adipose tissue, brain, heart, intestine, kidney, liver, lung, skeletal muscle, stomach, and trachea). Using this targeted MPS assay, the Javan et al. (2020) study assessed postmortem mammalian liver samples from 27 corpses from Italy and United States at various times since death (approximately 3.5 h–37 days). From this analysis of postmortem liver samples, the targeted panel demonstrated the ability to successfully detect gene expression signatures of mRNA exposed up to 37 days of autolysis, which can be used to confirm the putative identity of hepatic tissue fragments.

The questions of “how, why, and how long do some genes reactivate after organismal death?” are questions that have garnered a surge of research studies in the field of gene expression after death. Postmortem gene expression is a PI3K-Akt signaling-specific repetition of an evolutionary processes of development pathways where induction of a condition within the host body during the period of irreversible decline in functional status prior to death causes cells to struggle to survive. These survival efforts transpire by the cells altering their transcriptional processes to cause upregulation of developmental pathways which involves PI3K-Akt signaling (Dachet et al., 2021). Notably, protein kinase B (PKB, or Akt) regulates the activation of cell growth, metabolism, proliferation, and survival (Xie et al., 2019). This activation is operated by a multifactorial process that includes phosphoinositide-3-kinase (PI3K), a central enzyme in growth signaling pathways.

Studies have been undertaken to determine if PI3K-Akt signaling pathway is a recapitulation of development and/or a return to cellular stemness. The stemness of a cell is signified in its propensity for differentiation and self-renewal. Stem cells may attempt to maintain homeostasis between proliferation, quiescence, and regeneration via interfaces during fresh and bloat stages of postmortem. Additionally, the role of hypoxia inducible factor 1 subunit alpha (HIF1α) in this process has been considered (Semenza, 2012). Studies have linked the balance of HIF1α, caused by hypoxic pressure affecting acute stressors during the perimortem period, to stimulating the rejuvenating process in vertebrate tissues. The lack of oxygen occurs when the heart stops and hemoglobin no longer deliver oxygen to the tissues which triggers the efflux of intracellular fluids that causes coordinated proteolytic decay of organs.

Reports have associated the initiation of apoptosis in human cells at the phase of death as cells struggle to persist. Genetic studies of key regulators of apoptosis using liver tissues from autopsied samples, showed substantial down-regulation of the expression of anti-apoptotic functional gene groups and an up-regulation of pro-apoptosis genes for up to 4 days (Javan et al., 2015). Specifically, RNA expression levels of *BNIP3L* (anti-apoptotic), *CASP1* (anti-apoptotic), *CRADD* (anti-apoptotic), *DFFA* (pro-apoptotic), and *DIABLO* (pro-apoptotic) were reduced. *AIFM1* (pro-apoptotic), *CASP3* (pro-apoptotic), *CASP7* (pro-apoptotic), *FADD* (anti-apoptotic), and *XIAP* (anti-apoptotic) gene expressions increased. We hypothesize that these divergent conclusions are potentially due to mechanisms to achieve homeostatic equilibrium between the pro- and anti-apoptotic signals that are activated or decreasing in activity in decaying tissues to maintain cell survival. The expression of the anti-apoptotic gene *XIAP*, the most potent natural inhibitor of apoptosis, was greater than 28-fold after organismal death. In a related postmortem gene expression analysis of Italian cadaver prostate tissue, there was a marked up-regulation of mRNA transcripts that were still detectable up to 5 days after death. This study also demonstrated increased expression of *XIAP* and other pro-survival genes such as *BAG1* and *BCL2* (Tolbert et al., 2018).

## 4 Human microbiome

### 4.1 Human antemortem microbiome

In the past two decades, research of the human antemortem microbiome has extended from inceptive culture-based research of the oral and gut microbiome to molecular profiles of the microenvironments in a wide array of mucosal niches in the human body. The composition and activity of bacteria that form symbiotic relationships with the living human host perform necessary roles for food metabolism (De Vos et al., 2022), immune system homeostasis (Zheng et al., 2020), and reduction of pathogen proliferation (Coyte and Rakoff-Nahoum, 2019). Breakthroughs in DNA sequencing technologies by the Human Microbiome Project (HMP) have resulted in a change in basic assumptions in our understanding of cells in humans (The Integrative H (iHMP) Research Network Consortium, 2019). The HMP demonstrated that healthy adults contain about 10 trillion human cells, but as many as a debatable 10–100 trillion microbial cells depending on the location (Turnbaugh et al., 2007; Hamady and Knight, 2009; Sender et al., 2016). The project found that there are between 3,500 and 35,000 species-level operational taxonomic units (OTUs) representing about 600 genera (Human Microbiome Project Consortium, 2012).

It is important to note that before humans are born, they are essentially “microbial-cell free” (Sender et al., 2016). Once they pass through the uterus, the newborn receives its initial inoculum of microbes. As infants consume food and interact with parents/siblings and the environment, they become inoculated with microbes that live and thrive in their bodies. Gut microbiome divergence materializes at approximately 3 months of age and remains throughout childhood (Mallott et al., 2023). Bacteria exist in the intestines without deleterious consequences, and the gut epithelium typically is a barrier to microbial infiltration into the circulatory system. However, studies have shown that the entry of pathogens occurs when dendritic cells penetrate the intestinal epithelium and absorb bacteria from the intestinal lumen (Rescigno et al., 2001). Bacteria migrate through the human body as they are transported to distal lymphoid organs (Rodríguez et al., 2015) and are disseminated and colonize host niches of the blood, central nervous system, and urinary tract, which results in infection. Human-associated microorganisms have the potential to synthesize numerous sequence-specific DNA-binding factors that may mediate important host-microbe and microbe-microbe gene functions during life. Importantly, cohort data of the taxonomic and functional profiles of specific bacteria of the human microbiome have been used to assess disease and cause-specific mortality risks (Salosensaari et al., 2021).

The evolution of next-generation technologies has led to the question: “what is next for human antemortem microbiome studies?” The next phases will include improved consideration of microorganisms and their crucial roles in host health and disease. Elucidation of these roles is essential for breakthroughs and implementation of approaches for the mechanistic diagnosis of disease and the subsequent treatments. Artificial intelligence (AI) will be assessed in assisting the medical field with processing the massive inundation of data generated from microbiome functional analyses and host-microbiome interaction studies. AI transfer learning research, using a microbiome-based random forest model, was conducted as a diagnosis tool, and the results demonstrated that the technique was able to detect a broad spectrum of cancer types using blood samples (Xu et al., 2023). Huang et al. (2023) developed an AI machine learning approach that examined colony morphology and genomic data through an open-source high-throughput robotic strain isolation platform for the rapid generation of cultured biobanks.

### 4.2 Human postmortem microbiome

Death stops the mammalian biological clock, but the microbial one keeps ticking. It happens in a stepwise manner and is a time-dependent process. During the stages of decomposition, microorganisms migrate and adjust almost immediately due to hypoxia and the failure of the immune system. This sequence is relatively fast, and bacteria infiltrate the vertebrate liver in less than 3.5 h then reach the heart and other organs within 58 h (Javan et al., 2017). Anaerobic bacteria, such as *Clostridium* spp. in the liver, can double in less than 7.4 min (Willardsen et al., 1979; Javan et al., 2017).

**FIGURE 2:**
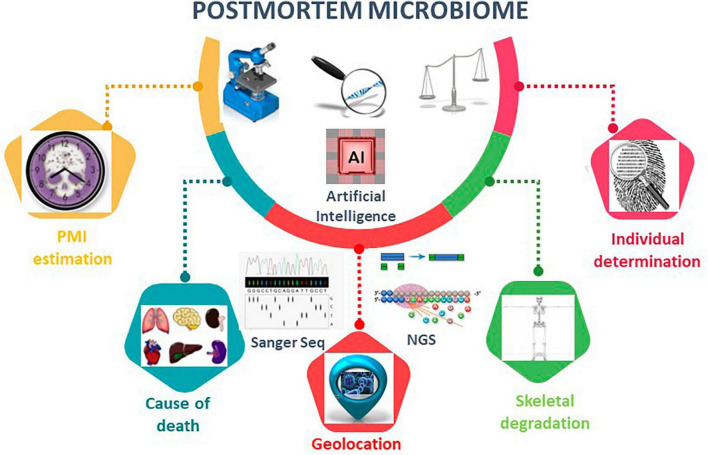
Postmortem microbiome diversity analysis using microbiome tools. Overview of the applications of postmortem microbiome in PMI estimation, cause of death, geolocation, skeletal degradation, and individual determination. Artificial intelligence, AI, is an emerging method to construct robust algorithms to analyze large postmortem microbial-based datasets. AI techniques leverage regression analyses, deep-learning artificial neural network models, and machine learning random forest to make predictions for the listed postmortem microbiome characterizations.

The microbiome of death is defined as microbial succession in the internal organs (e.g., brain, heart, liver, spleen) of decomposing humans and can provide physical and molecular evidence concerning interactions between putrefactive microorganisms and their mammalian hosts (Hyde et al., 2013, 2015; Can et al., 2014; Damann et al., 2015; Javan et al., 2016a,b, 2017; Metcalf et al., 2016; Pechal et al., 2018; Lutz et al., 2020; Figure 2). It is a long-held belief that mammalian internal organs are sterile in living hosts (Ford, 1901; Stewart, 2012). Thus, microbes discovered in internal organs of vertebrate remains may represent those that are directly associated with decomposition. Furthermore, from an evolutionary perspective, it would be interesting to investigate if there is a relationship between microbes and the mammalian species being decomposed by these microbes because it might indicate a “postmortem co-evolution.”

The current body of literature regarding mammal decomposition processes and microbial community assembly reveals distinct postmortem microbiome of human hosts. Microbial survey of internal organ tissues (e.g., brain, heart, liver, and spleen) of cadavers, associated with a homicide, suicide, overdose, and accidental death cases, demonstrated that the obligate anaerobe, *Clostridium*, was found in cadavers of varying PMIs, while the facultative anaerobe, *Lactobacillus*, was more abundant in cadavers with shorter PMIs. A second study performed an exploratory analysis of bacteria present in mouth and rectal scrapings taken at the onset and end of the bloat stage of corpses decomposing in a natural setting (Hyde et al., 2013, 2015). However, internal organs were not sampled in this study. In a related study to determine the microbial colonization of bone as an important mechanism of postmortem skeletal degradation, Damann et al. (2015) analyzed V3 region of the 16S rRNA gene from lower rib bones of 12 corpses and three gravesoil samples using bacterial sequencing. It was determined that Actinobacteria and Acidobacteria were more ubiquitous in the dry remains and gravesoil samples than in the advanced and putrid dry remains stages.

In a study involving microbial, metabolic, and soil biogeochemical analyses, Metcalf et al. (2016) tested the skin and gravesoil associated with four decaying human bodies for 82 days to ascertain the processes regulating microbial fingerprints during decomposition on different soil types. The results demonstrated a statistically reliable microbial succession among corpses that were sampled in the same season. Furthermore, PMIs were accurately determined across seasons and host species (compared to a mouse model). The study also revealed that the relative abundance of genes for amino acid degradation and hyperammonemia in proliferating bacteria increased (Metcalf et al., 2016). In a related study, a large-scale survey by Pechal et al. (2018) from 188 forensic cases representing an industrial-urban population demonstrated a positive correlation between cell motility and PMI. Also, the study showed that during putrefaction processes, the body provides robust niche differentiation among various body sites.

In a sex-related postmortem microbiome study, different tissues revealed differential associations between microbial signatures (Lutz et al., 2020). The reproductive organs from 40 European cadavers with PMIs ranging from 1 to 18 days, for example, showed that the prostate and uterus were increased with Clostridiales and Lactobacillales bacteria and decreased with ME1-12 bacteria compared to non-reproductive organs.

Traditional methods for determining PMI are based on postmortem facts, gravesoil microbial signatures, and metabolic and biochemical changes (Finley et al., 2016a,b; Thomas et al., 2017). However, AI-based microbiome analyses for PMI estimation are emerging topics in the elucidation of time since death (Table 2). In the Javan et al. (2016a) study, AI machine learning analysis was performed to determine correlations between PMI and an inventory of microbial taxa using random forest analysis. The results identified a list of candidate microbial succession that changed in abundance over time across sex and organ types. Specific bacteria were potentially predictive of precise points of the stages of decomposition. For instance, *Clostridium novyi* was relatively more prevalent at the longer times after death; however, an unknown *Clostridium* sp. was more abundant during early stages of decomposition. These host–microbe functions within putrefactive biomass conduct the efficient implementation of the Postmortem Clostridium Effect (PCE) which establishes the ubiquitous and putrefactive nature of *Clostridium* species in human decomposition (Javan et al., 2017).

**TABLE 2:** Prediction of PMI using AI application on postmortem microbiome.

Type of model	PMI/ADD	AI model	Sample location	References
Human	10 days	RF	Blood, brain, buccal cavity, heart, liver, spleen	Javan et al., 2016a
Human	500 ADD	RA	Ear canal, nasal cavity	Johnson et al., 2016
Human and Rat	30 days	RA	Gut	Li et al., 2023
Human	6 days	ANN	Vitreous humor	Bocaz-Beneventi et al., 2002
Human	3 days	RF	Mouth, rectum	Kaszubinski et al., 2020
*Sus scrofa* bones	5200 ADD	RF	Rib, scapulae	Randall et al., 2021
*Sus scrofa* bones	6322 ADD	RF	Rib, scapulae	Cartozzo et al., 2021
*Sus scrofa*	5 days	RF	Buccal cavity, skin	Pechal et al., 2014
Rat	59 days	RF	Oral cavity	Zhao et al., 2022
Mice	14 days	RF	Intestinal	Zhang et al., 2022
Mice	48 days	RF	Skin	Metcalf et al., 2013
Mice	15 days	RF	Cecum	Liu et al., 2020

RF, random forest analysis; RA, regression analysis; ANN, artificial neural networks.

## 5 Conclusion

It is crucial for the scientific community to engage in research to assess the complexity of human death and its physiological, transcriptomic, and microbiological implications. The studies cited in this review unveil the massive progress in elucidating the implications for human putrefaction. Death is inevitable; however, the study of the biological changes, gene expression, and microbial succession provide a missing piece of the postmortem enigma. The utilization of AI is poised to shed new light for computational systems able to simulate human-like cognition specifically for the analysis of big data to formulate accurate and robust PMI predictive algorithms.

In the future, the following research questions will be addressed: What are the successional changes in bacterial communities and in eukaryotic mRNA integrity levels that indicate “postmortem co-evolution after organismal death?” Our group’s goal is to provide the scientific community with free, publicly available information on postmortem abundances of RNA across biological conditions such as diverse tissue types and geographical factors. To achieve this goal, our project involves the creation of a Postmortem Gene Atlas (PGA) via data curation and analyses. We assert that this project will reveal new scientific information on how microbial populations and gene expression change in different organs as a function of time as a mammalian decays.

## Author contributions

KS: Writing – review and editing. SF: Writing – review and editing, Conceptualization, Investigation, Writing – original draft. CS: Writing – review and editing. RG: Writing – review and editing. GJ: Conceptualization, Investigation, Writing – original draft, Writing – review and editing, Funding acquisition.
